# Effects of adjunct testosterone on cardiac morphology and function in advanced cancers: an ancillary analysis of a randomized controlled trial

**DOI:** 10.1186/s12885-019-6006-5

**Published:** 2019-08-07

**Authors:** Jessica M. Scott, E. Lichar Dillon, Michael Kinsky, Albert Chamberlain, Susan McCammon, Daniel Jupiter, Maurice Willis, Sandra Hatch, Gwyn Richardson, Christopher Danesi, Kathleen Randolph, William Durham, Traver Wright, Randall Urban, Melinda Sheffield-Moore

**Affiliations:** 1 Department of Medicine, Memorial Sloan Kettering Cancer Center, New York, NY USA; 2 Department of Internal Medicine, The University of Texas Medical Branch, Galveston, TX USA; 3 Department of Anesthesiology, The University of Texas Medical Branch, Galveston, TX USA; 4 Department of Otolaryngology, The University of Texas Medical Branch, Galveston, TX USA; 5 Department of Preventive Medicine and Community Health, The University of Texas Medical Branch, Galveston, TX USA; 6 Department of Radiation Oncology, The University of Texas Medical Branch, Galveston, TX USA; 7 Department of Gynecologic Oncology, The University of Texas Medical Branch, Galveston, TX USA; 8 Department of Health and Kinesiology, Texas A&M University, 155 Ireland St., College Station, TX TX 77845 USA

**Keywords:** Testosterone, Cardiac function, Cachexia

## Abstract

**Background:**

Adjunct testosterone therapy improves lean body mass, quality of life, and physical activity in patients with advanced cancers; however, the effects of testosterone on cardiac morphology and function are unknown. Accordingly, as an ancillary analysis of a randomized, placebo-controlled trial investigating the efficacy of testosterone supplementation on body composition in men and women with advanced cancers, we explored whether testosterone supplementation could prevent or reverse left ventricular (LV) atrophy and dysfunction.

**Methods:**

Men and women recently diagnosed with late stage (≥IIB) or recurrent head and neck or cervical cancer who were scheduled to receive standard of care chemotherapy or concurrent chemoradiation were administered an adjunct 7 week treatment of weekly intramuscular injections of either 100 mg testosterone (T, *n* = 1 M/5F) or placebo (P, *n* = 6 M/4F) in a double-blinded randomized fashion. LV morphology (wall thickness), systolic function (ejection fraction, EF), diastolic function (E/A; E’/E), arterial elastance (Ea), end-systolic elastance (Ees), and ventricular-arterial coupling (Ea/Ees) were assessed.

**Results:**

No significant differences were observed in LV posterior wall thickness in placebo (pre: 1.10 ± 0.1 cm; post: 1.16 ± 0.2 cm; *p* = 0.11) or testosterone groups (pre: 0.99 ± 0.1 cm; post: 1.14 ± 0.20 cm; *p* = 0.22). Compared with placebo, testosterone significantly improved LVEF (placebo: − 1.8 ± 4.3%; testosterone: + 6.2 ± 4.3%; *p* < 0.05), Ea (placebo: 0.0 ± 0.2 mmHg/mL; testosterone: − 0.3 ± 0.2 mmHg/mL; p < 0.05), and Ea/Ees (placebo: 0.0 ± 0.1; testosterone: − 0.2 ± 0.1; p < 0.05).

**Conclusions:**

In patients with advanced cancers, testosterone was associated with favorable changes in left ventricular systolic function, arterial elastance, and ventricular-arterial coupling. Given the small sample size, the promising multisystem benefits of testosterone warrants further evaluation in a definitive randomized trial.

**Trial registration:**

This study was prospectively registered on ClinicalTrials.gov (NCT00878995; date of registration: April 9, 2009).

**Electronic supplementary material:**

The online version of this article (10.1186/s12885-019-6006-5) contains supplementary material, which is available to authorized users.

## Background

Cancer cachexia is a complex, multifactorial syndrome characterized by a progressive loss of skeletal muscle mass with or without loss of fat mass that cannot be fully reversed by conventional nutritional support [1]. Cachexia occurs in 50 to 80% of advanced cancer patients and is associated with decreased mobility [2], reduced response to chemotherapy [3], and is estimated to directly account for more than 20% of cancer-related deaths [2]. There are no established therapies for cancer cachexia; accordingly, identification and testing of effective interventions are of major clinical importance in this at-risk population.

Cancer cachexia involves not only the loss of skeletal muscle, but also results in pathologic alterations within the heart [4, 5]. The first report linking tumor burden and cardiac atrophy was first published in 1904 [6], and was extensively outlined using autopsies by Hellerstein and Santlago-Stevenson in 1950 [7]. More recent preclinical findings indicate that cardiac muscle loss occurs to a similar degree as in skeletal muscles, with concomitant impairment in systolic and diastolic function [8, 9]. Collectively, the global nature of cachexia portends the requirement for multifactorial treatment strategies with the capacity to augment or reverse whole-organism atrophy.

Testosterone therapy has been used in patients exposed to atrophic stimuli [10] to increase muscle strength and bone mineral density [11, 12]. The heart is also a target organ for steroids; there are receptors with a high affinity for testosterone in cardiomyocytes [13], suggesting that testosterone supplementation may also improve cardiac morphology and function. In support, a meta-analysis of randomized placebo-controlled studies found that testosterone administered to patients with chronic heart failure reduced systemic vascular resistance and increased both cardiac output and overall exercise capacity [14]. However, whether there are similar salutary cardiovascular effects of testosterone in patients with advanced cancers is not known. Accordingly, as an ancillary analysis of a randomized, placebo controlled trial investigating the efficacy of testosterone supplementation on body composition in men and women with advanced cancers [15], we explored whether testosterone supplementation could prevent or reverse left ventricular (LV) atrophy and dysfunction.

## Methods

### Patients and study design

Details of the design, rationale, and primary results of study have been published elsewhere [15]. This is an ancillary analysis of a RCT (NCT00878995) among men and women with histologically-confirmed advanced or recurrent squamous cell carcinoma of the cervix (stages IIB, IIIA, and IIIB) or head and neck squamous cell carcinoma (stage III or IV) conducted at the University of Texas Medical Branch at Galveston, TX. Major eligibility criteria were: [1] loss of at least 5% of body mass over the past 12 months, [2] Eastern Cooperative Oncology Group score of 0 or 1, [3] score of > 23 points on the 30 point Mini Mental State Examination. All study procedures were reviewed and approved by the institutional review board. Participation in both intervention groups continued for a maximum of 7 weeks or until unacceptable toxicity or withdrawal of consent, whichever came first. Patients were randomly allocated in blocks of three to receive weekly injections of either 100 mg of testosterone enanthate (*n* = 10) or placebo (*n* = 14). Interventions were matched in terms of setting (clinic-based), and length (7 weeks). All outcomes were evaluated at pre-randomization (study treatments were initiated ≤14 days) and were repeated within ≤7 days of the final treatment session at postintervention (month 3).

### Intervention

A testosterone replacement paradigm commonly used to treat hypogonadal men was chosen to include weekly intramuscular injections of either 100 mg testosterone enanthate or placebo (sterile saline) over a period of 7 weeks. Testosterone and placebo injections were given by a nurse using an opaque syringe to obscure visual differences between testosterone and placebo.

### Cardiac structure and function

Patients underwent two-dimensional transthoracic and pulsed Doppler imaging by use of a commercial ultrasound system (iE33, Phillips Healthcare). Images were obtained by one experienced sonographer in the long axis, short axis, and apical 4 chamber views according to the American Society of Echocardiography guidelines [16] to determine LV wall thickness, end-diastolic volume (EDV), end-systolic volume (ESV), and LVEF. LV volumes were calculated using the biplane Simpson method. Pulsed Doppler recordings were employed to assess diastolic filling; in particular, early (E) and atrial (A) peak mitral inflow velocities were measured and the ratio of early to late diastolic filling velocity (E:A) was calculated. Tissue Doppler data were used to assess mitral annular velocity (E’). The ratio of E/E’ was also used to assess diastolic function. Images were analyzed off-line by experienced technicians blinded to group allocation. A minimum of three consecutive cardiac cycles were measured and averaged.

End-systolic pressure (ESP) was calculated as 0.9 × brachial systolic blood pressure, a noninvasive estimate that accurately predicts LV pressure-volume loop measurements of ESP [17]. End-systolic elastance (Ees) was calculated as Ees = ESP/ESV, effective arterial elastance (Ea) was calculated as Ea = ESP/SV, and ventricular-vascular coupling was determined as Ea/Ees [18]. Systemic vascular resistance (SVR) was calculated as mean arterial pressure/CI × 80.

### Statistical analysis

Repeated-measures ANOVA was initially used to compare means between groups. Because of the small sample size and large amount of variability in the data, nonparametric tests were carried out at each level of intensity and at each time of measurement. Comparisons among groups were performed using the Kruskal-Wallis test. When differences were determined to be significant, pairwise comparisons were made using the Mann-Whitney method. The association between baseline cardiac morphology and function and change with testosterone was explored with Pearson correlation coefficient. Values are means ± SD; significance level was set at 0.05.

## Results

### Patient characteristics

Men and women recently diagnosed with late stage (IIB or higher) or recurrent head and neck or cervical cancer who were scheduled to receive standard of care chemotherapy or chemoradiotherapy were recruited to participate. A total of 28 potentially eligible patients were contacted for the study, and 24 (86%) were randomly grouped and administered an adjunct 7 weeks regimen of weekly intramuscular injections of either 100 mg testosterone or placebo. Of these, 16 (67%) completed cardiac assessments (testosterone, *n* = 1 M/5F; placebo, *n* = 6 M/4F). No significant differences were found in the baseline characteristics between placebo and testosterone groups (Table 1).

**Table 1:** Demographic and Treatment Characteristics of the Participants

Characteristic	All Patients (*n* = 16)	Placebo (*n* = 10)	Testosterone (*n* = 6)	*P* = value
Time (mos) from diagnosis to enrollment – mean (SD)	3.1 (3.2)	2.9 (3.4)	3.6 (3.1)	0.684
Age (yrs) – mean (SD)	50.9 (9.5)	48.4 (10.9)	55.0 (5.1)	0.189
BMI (kg/m^2^) – mean (SD)	22.1 (6.8)	23.9 (7.3)	19.3 (5.2)	0.200
^a^Exercise behavior (activity score) – mean (SD)	9.0 (8.0)	9.9 (9.6)	7.1 (3.6)	0.588
Race – no. (%)				0.330
Non-Hispanic white	11 (69)	6 (60)	5 (83)	
Other group	5 (31)	4 (40)	1 (20)	
Sex – no. (%)				0.091
Male	7 (44)	6 (60)	1 (17)	
Female	9 (56)	4 (40)	5 (83)	
Smoking – no. (%)	n = 16	n = 10	n = 6	0.355
Never	4 (25)	3 (30)	1 (17)	
Former	7 (44)	3 (330)	4 (67)	
Current	5 (31)	4 (40)	1 (17)	
Disease stage – no. (%)	*n* = 15	*n* = 9	*n* = 6	0.852
IIB	1 (7)	0 (0)	1 (17)	
III	0 (0)	0 (0)	0 (0)	
IIIB	4 (27)	3 (33)	1 (17)	
IV	0 (0)	0 (0)	0 (0)	
IVA	8 (53)	5 (56)	3 (50)	
IVB	2 (13)	1 (11)	1 (17)	
Cancer Type – no. (%)				1.000
Cervical	6 (38)	4 (40)	2 (33)	
Head/neck	10 (62)	6 (60)	4 (37)	
PEG feeding tube – no. (%)	6 (38)	3 (30)	3 (50)	0.986
Current Therapy – no. (%)				
Chemotherapy	11 (69)	6 (60)	5 (83)	0.985
Radiotherapy	13 (81)	9 (90)	4 (67)	
Other Therapy	0 (0)	0 (0)	0 (0)	
Prior therapy – no. (%)	*n* = 16	*n* = 10	*n* = 6	0.927
Surgery	2 (13)	2 (20)	0 (0)	
Chemotherapy	0 (0)	0 (0)	0 (0)	
Radiotherapy	0 (0)	0 (0)	0 (0)	
Other Therapy	0 (0)	0 (0)	0 (0)	
Current Medications – no. (%)	*n* = 16	*n* = 10	*n* = 6	0.728
Beta-blocker	0 (0)	0 (0)	0 (0)	
ACE inhibitor	1 (6)	1 (10)	0 (0)	
ARB	1 (6)	1 (10)	0 (0)	
Diuretic	1 (6)	1 (10)	0 (0)	
Calcium channel blocker	1 (6)	1 (10)	0 (0)	
Aspirin	3 (19)	2 (20)	1 (17)	
Statin	2 (13)	2 (20)	0 (0)	
Pre-existing conditions – no. (%)	*n* = 16	*n* = 10	*n* = 6	0.586
Peripheral vascular disease	2 (13)	1 (10)	1 (17)	
Coronary artery disease	1 (6)	1 (10)	0 (0)	
Osteoporosis	1 (6)	0 (0)	1 (17)	
Arrhythmia	0 (0)	0 (0)	0 (0)	
Arthritis	0 (0)	0 (0)	0 (0)	
Type II diabetes	2 (13)	2 (20)	0 (0)	
Hyperlipidemia	2 (13)	2 (20)	0 (0)	
Hypertension	1 (6)	1 (10)	0 (0)	

Abbreviations: *SD* standard deviation, *BMI* body mass index, *ACE* angiotensin converting enzyme, *ARB* angiotensin II receptor blockers. ^a^Exercise behavior sum of mild, moderate, and strenuous exercise obtained from ActiGraph 3 axis accelerometry monitors available in a subset of patients (*n* = 8 placebo; *n* = 4 testosterone). No significant differences between the groups. *P*-values provided are from t-tests when group means were compared or chi-square tests when comparing frequency of cases between the groups

### Testosterone supplementation

Pre-study average total serum testosterone levels were significantly different between males and females (328 ± 420 ng/dL and 17 ± 14 ng/dL respectively, *p* < 0.001). Testosterone levels in females in the placebo group were unchanged from pre- (16 ± 9 ng/dL) to post-intervention (23 ± 24 ng/dL; *p* = 0.40) whereas testosterone levels were increased in the testosterone group (pre: 19 ± 17 ng/dL; post: 644 ± 327 ng/dL; *p* = 0.01). Testosterone levels in males in the placebo group decreased from 354 ± 193 ng/dL to 342 ± 174 ng/dL (*p* = 0.80). Only one male was randomized into the testosterone group; serum testosterone level increased from 177 to 885 ng/dL. Estrogen values remained below 62 pg/mL for all subjects and there were no changes in response to testosterone treatment.

### LV morphology, resting heart rate, and blood pressure

**Fig. 1:**
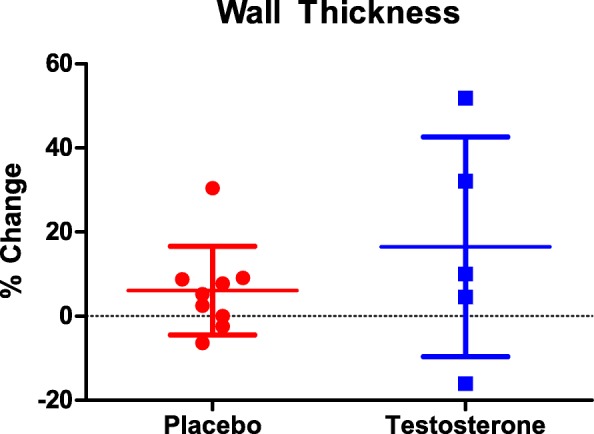
Percent change in left ventricular posterior wall thickness from pre to post-intervention in placebo (red) and testosterone (blue)

No significant differences were observed in LV posterior wall thickness in placebo (pre: 1.10 ± 0.1 cm; post: 1.16 ± 0.2 cm; *p* = 0.11) or testosterone group (pre: 0.99 ± 0.1 cm; post: 1.14 ± 0.20 cm; *p* = 0.22); Fig. 1. No differences between groups in change in resting heart rate (placebo: + 3 ± 11 bpm; testosterone: + 6 ± 11 bpm; *p* = 0.39) or mean arterial pressure (placebo: + 3 ± 12.1 mmHg; testosterone: − 5 ± 12.1 mmHg; *p* = 0.28) were observed. There was no significant correlation between baseline values and change in LV morphology (r = 0.48).

### LV volumes, systolic, and diastolic function

**Fig. 2:**
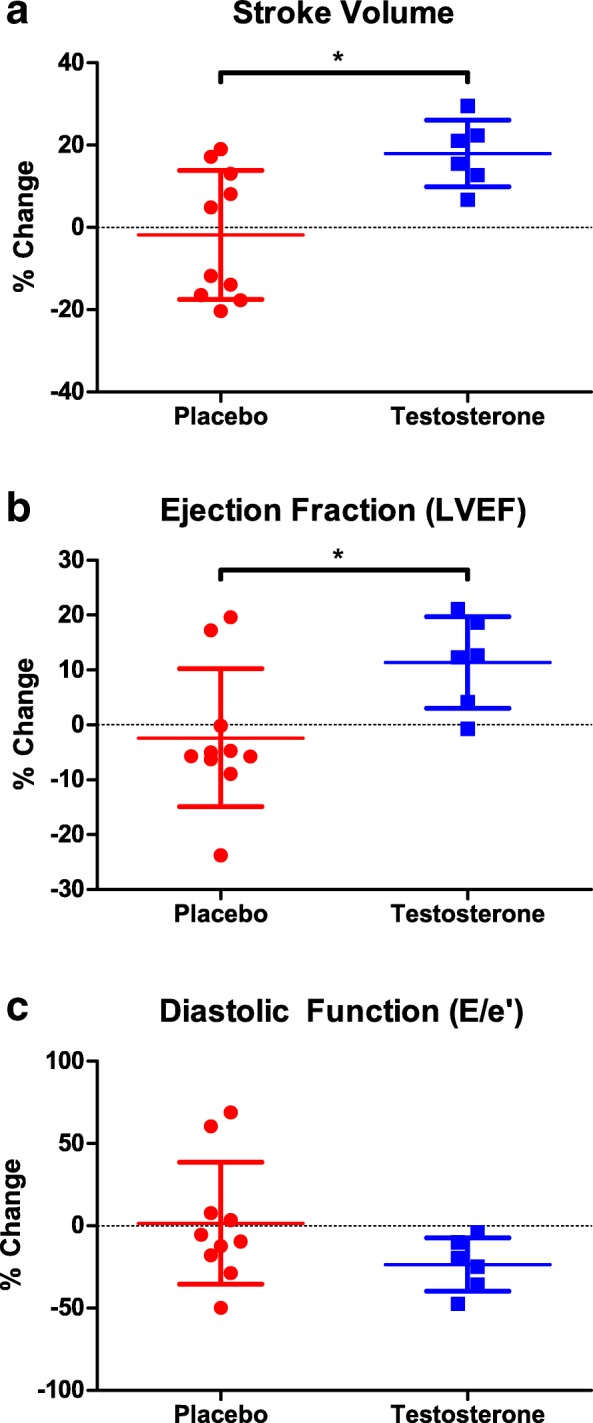
Percent change in stroke volume (**a**) left ventricular ejection fraction (**b**), and E/E’ (**c**) from pre to post-intervention in placebo (red) and testosterone (blue)

No differences in end diastolic volume (EDV) or end systolic volume (ESV) were observed in the placebo (EDV, pre: 118.9 ± 16.3 mL, post: 119.3 ± 16.5 mL; *p* = 0.95; ESV, pre: 46.9 ± 13.3 mL, post: 49.2 ± 8.2 mL; *p* = 0.62) or testosterone group, (EDV, pre: 109.5 ± 16.3 mL, post: 116.0 ± 16.5 mL; *p* = 0.16; ESV, pre: 46.2 ± 13.3 mL, post: 41.2 ± 8.2 mL; *p* = 0.18). There was a significant difference in change in stroke volume between the placebo (− 1.9 ± 5.3 mL) and testosterone (+ 11.5 ± 5.3 mL) groups (Fig. 2a). There was a significant difference in change in LV ejection fraction (LVEF) between the placebo (− 1.8 ± 4.3%) and testosterone (6.2 ± 4.3%) groups (*p* = 0.02) (Fig. 2b). There was a significant negative association between baseline and change in LV ejection fraction in the testosterone group (r = 0.95; *p* < 0.05). Diastolic function assessed by E/A (placebo pre: 1.1 ± 0.3 cm/s; post: 1.3 ± 0.4 cm/s; *p* = 0.35; testosterone pre: 1.1 ± 0.3 cm/s; post: 1.0 ± 0.4 cm/s; *p* = 0.63) and E/E’ (placebo pre: 6.0 ± 2.0; post: 5.7 ± 1.6; *p* = 0.75; testosterone pre: 7.7 ± 2.0; post: 5.7 ± 1.6; p = 0.63) (Fig. 2c) was preserved in both groups. Absolute changes in volumes, systolic, and diastolic function are presented in Additional file 1**.**

### Ventricular-vascular coupling

**Fig. 3:**
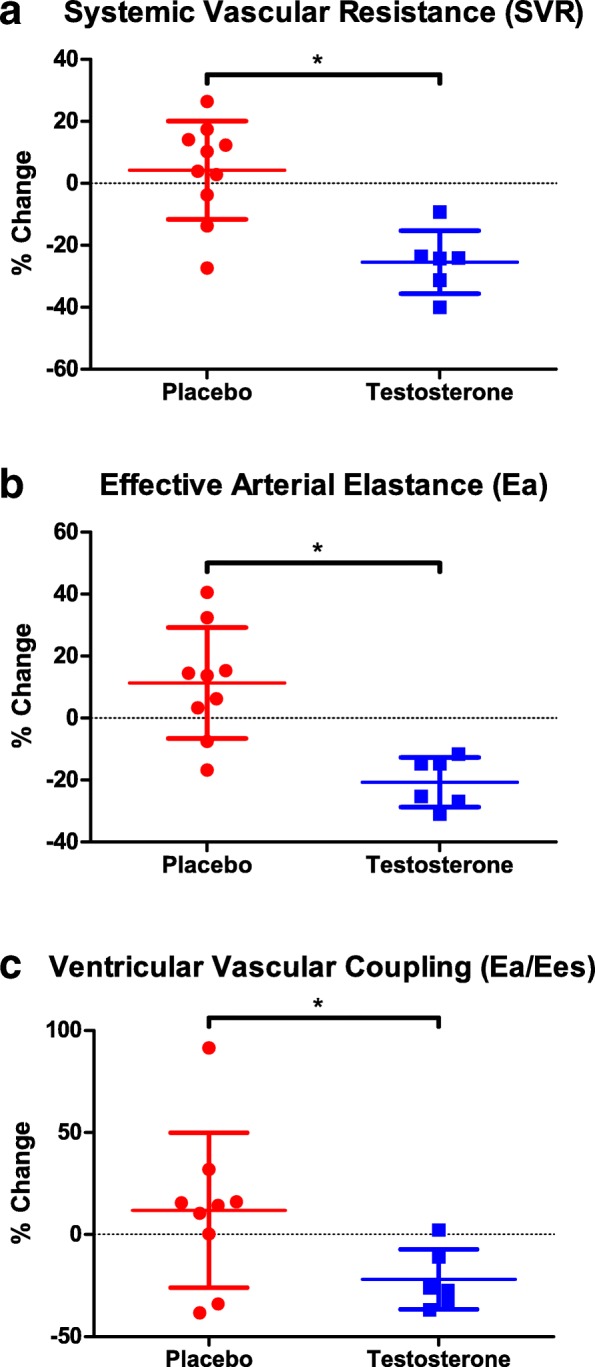
Percent change in systemic vascular resistance (**a**), arterial elastance (**b**), and ventricular-vascular coupling (**c**) from pre to post-intervention in placebo (red) and testosterone (blue)

End-systolic elastance (Ees) was unchanged in both groups (placebo pre: 2.4 ± 0.7 mmHg/mL; post: 2.4 ± 0.5 mmHg/mL; *p* = 0.79; testosterone pre: 2.4 ± 0.7; post: 2.4 ± 0.5; *p* = 0.85). There was a significant difference between groups in change in systemic vascular resistance (SVR, placebo: 45.7 ± 166.9 dynes/sec/cm^5^; testosterone: − 359.3 ± 166.9 dynes/sec/cm^5^; Fig. 3a), effective arterial elastance (Ea, placebo: 0.0 ± 0.2 mmHg/mL; testosterone: − 0.3 ± 0.2 mmHg/mL; Fig. 3b), and ventricular-vascular coupling (Ea/Ees, placebo: 0.0 ± 0.1; testosterone: − 0.2 ± 0.1; Fig. 3c). No significant associations were observed between baseline and change in ventricular-vascular coupling. Absolute changes in ventricular-vascular coupling are presented in Additional file 1**.**

## Discussion

This is the first randomized trial to explore the potential efficacy of testosterone to augment / reverse cardiac morphology and function in patients with advanced cancers. The major new findings of this study were that compared with placebo, testosterone improved LV systolic function, as well as ventricular-vascular coupling. This may have important health implications for patients with cachexia given that this entity has no established evidence-based interventions that improve outcomes.

Changes in cardiac morphology and function may stem from the cancer itself and/or the cardiotoxic effects of cancer therapies [19]. For instance, Springer et al. [8] reported extensive loss of cardiomyocyte volume and replacement with fibrotic tissue among patients who died of pancreatic, lung, and colorectal cancer; however, a subset of patients with significant cancer-related weight loss and cachexia had reduced LV wall thickness and mass compared with cancer patients without cachexia. A reduction in LV mass following anthracycline-based chemotherapy has also consistently been reported [20, 21] and is associated with major adverse cardiac events (cardiovascular death, appropriate implantable cardioverter-defibrillator therapy, or admission for decompensated HF) [21]. Of note, average BMI of included patients was ~ 27 kg/m^2^, and whether patients with cachexia were included was not reported [20, 21]. The present study confirms and extends previous reports by including patients with advanced cancers, none of whom had been previously treated with cytotoxic therapy or radiotherapy. Collectively, these findings indicate that cardiac alterations in patients with advanced cancers is part of a complex, systemic issue that results in widespread muscle wasting. Accordingly, intervention strategies with multifactorial effects will be required to reverse whole-organism atrophy.

At least 19 studies have assessed the efficacy of pharmacological agents in clinical trials to manage cancer cachexia [22]; however, few have explored the potential salutary effects on cardiac morphology and function. Testosterone therapy has been used in patients exposed to atrophic stimuli [10] to increase muscle strength and bone mineral density [11], and we previously reported that in patients with advanced cancer adjunct testosterone improved lean body mass and was associated with increased quality of life, and physical activity compared with placebo [15]. Previous findings from non-oncology settings indicate that exogenous testosterone may also directly induce physiological cardiac myocyte hypertrophy [23]. For instance, among men with type 1 diabetes, higher total testosterone was associated with higher LV mass and volume [24], and Subramanya and colleagues [25] recently reported that after a median of 9.1 years, higher free testosterone levels were independently associated with an increase in LV mass in women and men in the Multiethnic Study of Atherosclerosis. In RCTs, testosterone treatment improved cardiac biomarkers in patients with type II diabetes [26], and reduced systemic vascular resistance and increased both cardiac output and overall exercise capacity in heart failure patients [14]. Similar findings were observed here in patients with advanced cancers; compared with placebo, testosterone improved indices of LV function. In addition, patients with the lowest LV ejection fraction at baseline experienced the greatest improvement with testosterone, suggesting that testosterone may be an important intervention for patients with poor LV ejection fraction. Nevertheless, these findings should be interpreted with caution given the small sample size. Collectively, these findings indicate that testosterone supplementation may be an effective intervention to improve cardiac function; however, larger trials are needed to address whether testosterone is fully protective against cardiac atrophic remodeling in patients with advanced cancers.

The mechanisms underlying testosterone-induced cardioprotection are not fully known; however, may involve both cardiac and vascular systems. Cardiomyocytes contain receptors with a high affinity for testosterone [13] and in vitro studies of nonhuman cardiac myocytes found that testosterone can decrease action potential duration (thereby altering repolarization) and peak shortening times [27]. Testosterone is also an acute vasodilator [28] and lowers blood pressure [29]. Thus, understanding how the heart and systemic vasculature function independently as well as how they interact (termed ventricular-arterial coupling) is important when evaluating global cardiovascular function [17]. In the present study we found that testosterone had beneficial effects on vascular parameters (e.g., Ea, SVR), which in turn, improved ventricular-vascular coupling compared to placebo-treated patients. Future studies evaluating the mechanistic underpinnings of the effects of testosterone on cardiac and peripheral vasculature in the cachectic setting are needed.

In current clinical practice, the discipline of cardio-oncology traditionally focuses on the detection and management of cancer treatment-induced reductions in cardiac function (i.e., LVEF), and/or development of overt heart failure [30–32] and coronary artery disease [33]. Intriguingly, based on conventional metrics, all patients in the current study have ‘normal’ cardiac function (e.g., LVEF > 55%). Nevertheless, there is burgeoning interest in detection of early and subclinical therapy-related cardiac consequences, including changes in cardiac size and ventricular-vascular coupling. Furthermore, techniques such as assessing the heart during exercise has provided novel prognostic information beyond traditional resting cardiac measures in patients with breast cancer [34]. Collectively, these findings indicate that evaluating cardiac morphology and function in the cachectic setting, as well as evaluating other metrics such as cardiorespiratory fitness and cardiac function during exercise will be important in the design of future intervention trials. Given the systemic effects of cachexia, evaluation of multimodal approaches including nutritional support, pharmacological intervention, and exercise training will be important for this high-risk population.

A number of study limitations should be considered. First, the trial was designed to assess the effect of testosterone treatment on lean body mass, and changes in cardiac parameters were not predefined outcome measures. Second, our sample size was small. Trials with larger samples sizes are needed to definitively assess the efficacy of testosterone on cardiac morphology and function in advanced cancers. Third, our subject population was predominantly female, and although androgens stimulate skeletal muscle protein synthesis similarly between men and women [35], potential sex differences in cardiac androgen receptor density [36] and the mechanisms of response to testosterone treatment may limit the generalizability of our findings. For instance, following exercise training the development of LV hypertrophy and increase in cardiorespiratory fitness in females was markedly blunted compared with males [37]; whether females have blunted response to testosterone compared to males should be addressed in future studies. Finally, to fully characterize the physiological importance of atrophic remodeling and potential efficacy of testosterone supplementation, there is a need to move beyond the study of global measures of LV function at rest. For example, reduced strain and strain rate revealed impaired myocardial function prior to LVEF decline [38] in cancer patients treated with anthracycline-containing therapy. Thus, evaluation of cardiac and vascular function with advanced imaging techniques at rest [39], as well as responses to a peak cardiopulmonary exercise test [40], may provide important insight into characterizing the ‘cachectic heart’.

## Conclusions

In patients with advanced cancers, testosterone was associated with favorable changes in left ventricular systolic function, arterial elastance, and ventricular-arterial coupling. There are promising multisystem benefits of testosterone; however, given the small sample size in the current study, further evaluation in a larger randomized trial is warranted.

## Additional file

Additional file 1: Absolute change in cardiac outcomes. (PDF 42 kb)

## Data Availability

The datasets used and/or analysed during the current study are available from the corresponding author on reasonable request.
