# Supplementary material for: Effects of adjunct testosterone on cardiac morphology and function in advanced cancers: an ancillary analysis of a randomized controlled trial
Source: BMC Cancer. 2019 Aug 7;19:778. doi: 10.1186/s12885-019-6006-5 (PMC6686390; doi:10.1186/s12885-019-6006-5)
Supplement: Supplementary file 1 — Absolute change in cardiac outcomes. (PDF 42 kb) [file 12885_2019_6006_MOESM1_ESM.pdf]

Additional File 1. Absolute change in cardiac outcomes.

		PLACEBO			TESTOSTERONE					
		Mean		SD	Mean		SD	Group	Time	Group*Time
**SV**	PRE	72	±	7.0	63	±	4.8			
	POST	70	±	8.7	75	±	8.8	0.487	0.097	0.024
**EF**	PRE	0.61	±	0.06	0.58	±	0.06			
	POST	0.59	±	0.07	0.65	±	0.02	0.475	0.304	0.07
**Ea**	PRE	1.46	±	0.18	1.63	±	0.23			
	POST	1.60	±	0.24	1.28	±	0.16	0.323	0.181	0.004
**Ea/Ees**	PRE	0.66	±	0.17	0.73	±	0.18			
	POST	0.70	±	0.20	0.55	±	0.06	0.493	0.3	0.078
**SVR**	PRE	1236	±	179	1365	±	234			
	POST	1282	±	240	1006	±	154	0.342	0.047	0.012
**E/e'**	PRE	5.97	±	1.52	7.66	±	2.01			
	POST	5.74	±	1.59	5.74	±	1.64	0.173	0.087	0.175

SV, stroke volume; EF, left ventricular ejection fraction; Ea, end arterial elastance; Ea/Ees, ventricular vascular coupling; SVR, systemic vascular resistance
